# Research on Dynamic Performance of CRTSⅢ Type Slab Ballastless Track under Long-Term Service

**DOI:** 10.3390/ma15062033

**Published:** 2022-03-10

**Authors:** Zhiping Zeng, Yancai Xiao, Weidong Wang, Zhibin Huang, Wei Wei, Saidi Boumedienne Houdou

**Affiliations:** 1 School of Civil Engineering, Central South University, Changsha 410075, China; 203160@csu.edu.cn (Z.Z.); 214812219@csu.edu.cn (Y.X.); wd1997@csu.edu.cn (W.W.); csu0206@yeah.net (S.B.H.); 2 MOE Key Laboratory of Engineering Structures of Heavy Haul Railway, Central South University, Changsha 410075, China; 3 Southeast Coastal Railway (Fujian) Co., Ltd., Fuzhou 350001, China; 4 China Railway Eryuan Engineering Group Co., Ltd., Chengdu 610031, China; weiwei0204@yeah.net

**Keywords:** China Railway Track System type slab ballastless track (CRTSIII SBT), fatigue load, falling axle impact, parting, dynamic response

## Abstract

To fill the blank in the research on the dynamic performance of track structure under long-term service, the dynamic response study of China Railway Track System Ⅲ type slab ballastless track (CRTSIII SBT) under the action of fatigue for 30 million times and the parting between track slab and self-compacting concrete (SCC) was carried out. By establishing the finite element model of the CRTSIII SBT structure and taking the stiffness change of isolation layer and fastener under fatigue state and the parting during service as the research objects, combined with the full-scale model test, the dynamic response amplitude and vibration law of track structure was analyzed based on the finite element model of axle falling test method. The results show the following: (1) Under the fatigue load, the acceleration of rail and base increases obviously, the longitudinal tensile stress of SCC surface decreases, the longitudinal tensile stress of base surface increases, and the vertical stress of each layer of track structure increases as well. (2) Under the action of the parting, the dynamic response of each structural layer increases, and the change of acceleration and stress of each layer under the action point of axle falling is the most obvious. (3) The fatigue load will weaken the vibration damping performance of the track, and the parting will continue to develop under the action of the falling axle, resulting in partial or total failure of the SCC layer. Both of them will aggravate the dynamic response of the track structure and affect driving safety, which should be paid attention to during maintenance.

## 1. Introduction

With the rapid development of high-speed railways in recent ten years, high-speed railways play an increasingly important role in the whole railway transportation system with their characteristics of high speed and large capacity. They have become the inevitable choice and core technology of modern railway transportation and have strong competitiveness in medium-distance travel [1,2,3]. The technical requirements of high-speed railways for high smoothness, high stability, durability, and less maintenance have promoted the leap of the overall technology of railway track [4]. At present, the large-scale design, construction, and operation of China’s high-speed railway in the early stage have almost been completed, and the existing line has begun to enter the long-term stable operation stage. In order to ensure the highly safe, smooth, and stable operation state of the line, line maintenance and early defect warning are particularly important.

**Figure 1:**
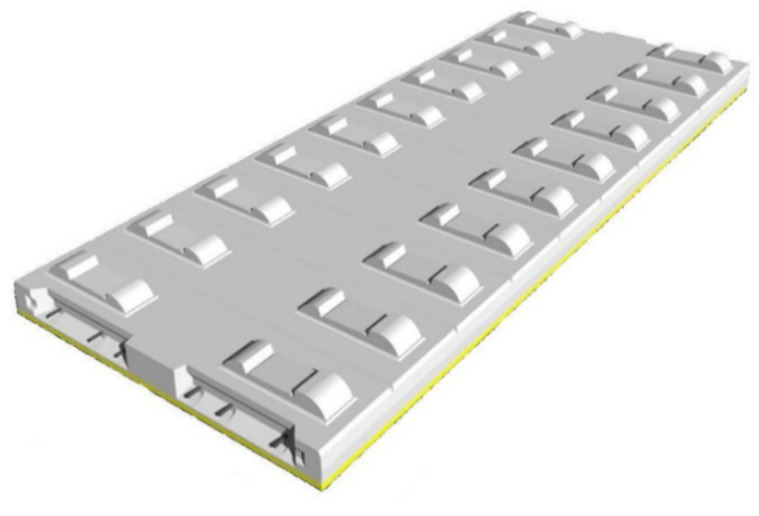
Schematic diagram of CRTSIII SBT.

In view of the existing slab (CRTSI type, CRTSII type) and double block (CRTSI type, CRTSII type) ballastless tracks with disadvantageous service conditions such as parting, slab end warping, track slab arch, and concrete cracking, China has formed the CRTSIII SBT system, which has been widely used in new high-speed railways, through continuous improvement and innovation [5]. CRTSIII SBT mainly draws on Borg slab track technology from Germany [6]. The track slabs are connected with each other through longitudinal (the direction along the rail) reinforcement at the end of the track slab, and the mortar adjustment layer is used as the bonding between the track slab and the base. The track structure is shown in Figure 1.

**Figure 2:**
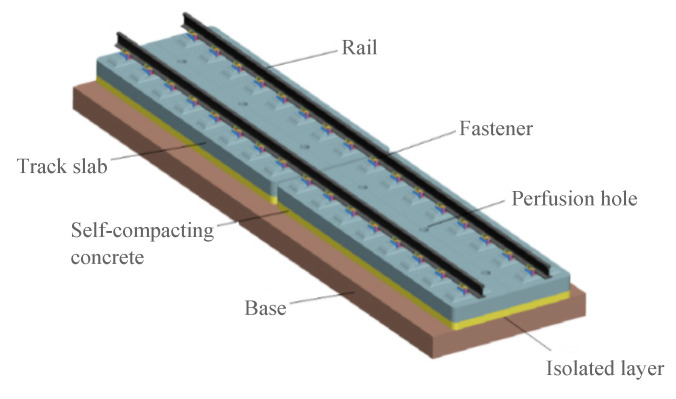
Schematic composite diagram of CRTSIII SBT.

CRTSIII SBT structure adopts a unit composite structure with reasonable stress, which is mainly composed of steel rail, fastener, precast track slab, SCC, U-shaped ribs, limit groove, intermediate isolation layer (geotextile or rubber cushion), and reinforced concrete base or support layer [7]. In the vibration reduction section, the geotextile can also be replaced with 20 mm thick ethylene propylene diene monomer (EPDM) microporous rubber vibration reduction cushion. The mature and reliable convex groove limit mode is selected for the limit mode of the unit slab, which improves the structural stability. The engineering material of the main structure is prestressed reinforced concrete, which ensures the durability of the structure. An isolation layer is set between the composite slab and the base, which can improve the stress of the structural system and have good maintenance performance. In terms of construction, the track slab is prefabricated in a factory and the SCC is poured on-site. The construction process is simple and the track structure has good environmental adaptability, being able to adapt to different climatic conditions such as warm, cold, and severe cold [8]. The track structure is shown in Figure 2.

In recent years, high-speed railways have played an increasingly important role in the whole railway transportation system. Countries have carried out relevant research and application of ballastless tracks [9,10,11,12,13]. For the CRTSIII SBT structure system, many scholars have also carried out much theoretical and experimental research on its static, dynamic, and fatigue properties. Zeng et al. established a fatigue finite element numerical model of the CRTSIII SBT structure system based on the three-stage model and focused on the fatigue characteristics of the CRTSIII SBT SCC under heavy load [14]. Based on the plastic damage theory of concrete, Cai et al. established the nonlinear damage finite element model of the CRTSIII, analyzed the deformation of track structure under different subgrade frost heave conditions, put forward the amplitude limit of subgrade frost heave [7]. Li et al. characterized the bonding interface characteristics under different plastic viscosities by calculating the fractal dimension and studied the effect of plastic viscosity on the mechanical properties between the SCC layer and steam curing concrete [15]. Ma et al. studied the rheological properties of the CRTSIII SBT SCC cementitious material containing superplasticizer, air-entraining agent, and defoamer and the effects of various additives on the transparency of cementitious material [16]. Through a series of full-scale model hammering tests, Zheng et al. studied the vibration characteristics and transmission law of ballastless track and analyzed the vibration reduction effect of rubber isolation layer [17]. Xu et al. proposed a method based on Gaussian curvature mode, which can be used to detect and accurately locate the internal damage of ballastless track [18]. Yang et al. established a three-dimensional calculation model considering geographical location and environmental conditions and analyzed the temperature characteristics of ballastless track under continuous high-temperature weather [19]. Luo et al. extended the classical vehicle-track coupled dynamics theory and thoroughly implemented track flexibility and advanced coupling relations into a three-dimensional train-slab track system [20]. However, up to now, the international research on the fatigue performance of the CRTSIII SBT structure system under long-term loads of tens of millions of times or more is still blank.

The structural layers of the CRTSIII SBT structure in service will have different mechanical property degradation or damage with the increase in service life and the complexity of the environment [21,22]. A model test is a research method in which a model similar to the prototype is used for experimental research, and the research results are applied to the prototype, which can control the main test parameters without being restricted and affected by environmental conditions [23,24,25]. However, to carry out model test research, in addition to arranging and embedding sensors on the surface and interior of tracks and subgrades, special loading devices are also required, especially dynamic loading devices that are often complicated. The axle falling impact device can easily apply impact loads to the track and roadbed, so the axle falling test has become one of the important ways to study the dynamic characteristics of the track and roadbed, and it is widely used in the research of various types of tracks [26]. In view of this, the finite element model of the CRTSIII SBT structure is established in this paper, which is compared with the measured data of fatigue static load test and axle falling test of CRTIII SBT in the existing literature to verify the reliability of the model. Furthermore, taking the stiffness change of isolation layer and fastener under fatigue state and the parting between track slab and SCC during service as the research object, the dynamic performance response of track structure under different working conditions is studied and analyzed based on the finite element model of the axle falling test method. This work is expected to provide a reference for the condition evaluation research and maintenance strategy formulation of CRTIII SBT.

## 2. Establishment and Verification of Finite Element Model of CRTIII SBT Structure

### 2.1. Establishment of a Finite Element Model

**Figure 3:**
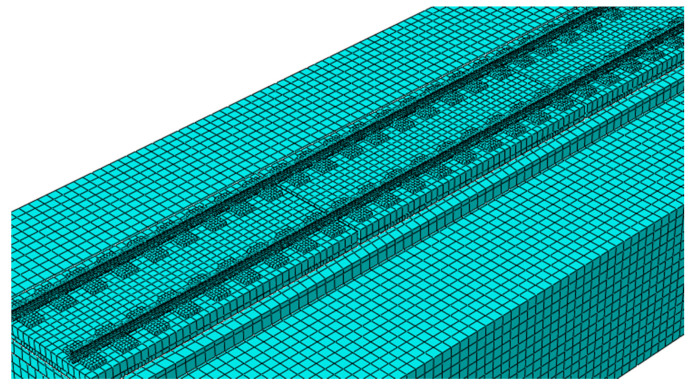
Finite element model of CRTSIII SBT.

The finite element model of the CRTSIII SBT structure system on subgrade includes wheelset, rail, fastener, track slab, U-shaped ribs, SCC layer, isolation layer (geotextile), elastic cushion, base, and subgrade bed. In order to eliminate the influence of boundary effect on the calculation results, the model arranges three unit CRTSIII SBT slabs on one base, with a total length of 16.94 m, as shown in Figure 3. The limit of the unit slab is realized by mutual embedding between the SCC layer and the base groove, and the elastic cushion is used as the buffer layer around the groove. The width of the slab gap between the two track slabs is 70 mm. The calculation results of the intermediate slab are taken for analysis and verification.

**Figure 4:**
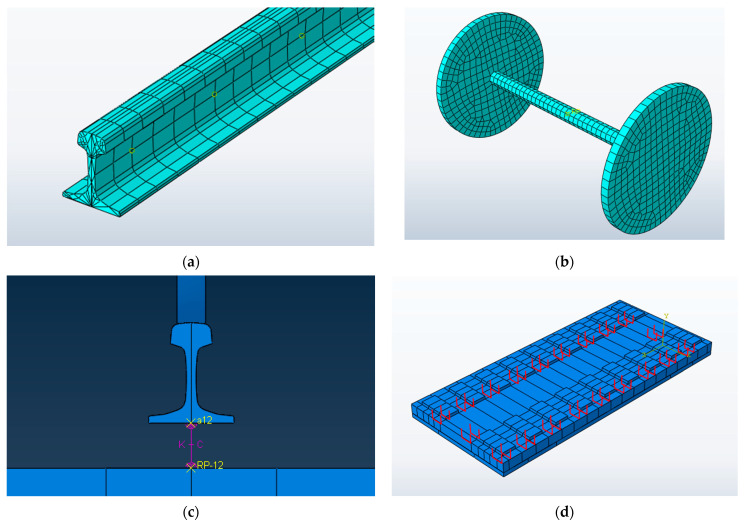
Finite element model of the main components: (**a**) rail finite element model; (**b**) wheelset finite element model; (**c**) finite element model of spring damping element; (**d**) finite element model of U-shaped ribs.

The model mainly considers and studies the mechanical characteristics of the CRTSIII SBT structure system and does not focus on the stress state of the wheelset; that is, the wheelset is simulated by a discrete rigid body. Since the model does not focus on the contact relationship between wheel and rail, the wheelset is simplified into a disc with equal mass and equal radius, as shown in Figure 4a. The rail is CHN60 rail (60 kg/m), which is simulated by a solid element, as shown in Figure 4b. The fastener uses a spring damping unit to connect the rail with the rail bearing platform. The connecting surface uses the size of the real under rail backing plate, and it uses the point-to-surface coupling method to establish the reference points for coupling with the rail bottom and rail bearing platform, respectively. The spring damping unit is used to connect the corresponding two reference points, and the stiffness and damping between the two areas are controlled through the reference point, so as to reduce the stress concentration at the fastener position, as shown in Figure 4c. As the connecting part between the track slab and the SCC layer, the U-shaped ribs are simulated by truss element, modeled according to the actual size, and connected with the track slab and the SCC layer by embedding, as shown in Figure 4d. P5600 track slab commonly used in the subgrade section is selected as the track slab, and solid units are used for the track slab, SCC layer, and base. For the part of the subgrade bed below the base, because the vertical (the direction perpendicular to the rail) stress of the subgrade bed needs to be calculated and analyzed in this paper, the part of the subgrade bed is also simulated by a solid element, which is divided into two parts: the surface layer and the bottom layer of the subgrade bed.

The meshing techniques are divided into three types: structured meshing, swept meshing, and free meshing. The difficulty of division decreases in turn, but the quality of the mesh also decreases [27]. Due to the different shapes of the parts of the CRTSIII SBT structure, in order to improve the calculation accuracy as much as possible and reduce calculation cost, the wedge sweep mesh is used for the rails, and the other parts are divided by the hexahedron structured mesh. Because the computer requires the surface–surface contact mesh to be divided into the main surface and the slave surface during analysis, if the mesh sizes of the master surface and the slave surface are similar, the calculation efficiency will be greatly reduced. Therefore, the larger surface should be used as the main surface, that is, mesh settings in the overall model should be dense on the top and sparse on the bottom to speed up the calculation [28]. Therefore, in terms of mesh size, the global seed spacing selected for this model is 50 mm for wheels, 40 mm for axles, 20 mm for rails, 60 mm for rail support, 150 mm for track slab, 150 mm for SCC, 200 mm for base, and 200 mm for subgrade. For parts such as rail arc chamfering and rail plate loading point, denser local seeds are defined to ensure calculation accuracy.

The contact mode of the model is defined according to the actual mechanical characteristics and contact state. In the finite element model of the CRTSIII slab ballastless track structure system, the track slab and the SCC are connected by binding, thereby enhancing the bonding strength between the interfaces. This is because in the on-site construction process, the SCC is poured on-site and the bond between it and the track slab is relatively tight, and a large number of U-shaped ribs are distributed between the two components to limit the position. In the contact simulation between the SCC and the isolation layer, and between the isolation layer and the base, the normal direction is selected as a hard contact with a specific stiffness, and the tangential direction is considered as a frictional contact with a friction coefficient of 0.8. The contact between the base and the subgrade also adopts normal hard contact and tangential friction contact. The bottom and all sides of the subgrade are fully restrained.

In the weighting algorithm of the model contact method, the selection of the master and slave surfaces of the contact surface also affects the calculation results to a certain extent. During the contact process of the two contact surfaces, it is necessary to judge whether the interface of the master control and the slave surface penetrates, which is generally detected by the constraint enhancement method. In the master–slave algorithm of the software, the nodes of the slave plane can be controlled to penetrate into the master plane, but the nodes of the master plane cannot be prevented from penetrating into the slave plane. Excessive penetration effects between the contact surfaces will affect the accuracy of the calculation results. Therefore, in the contact between the SCC and the isolation layer, and between the isolation layer and the base, the selection of the master and slave surfaces basically follows the following two principles: (1) The sparser mesh is used as the master surface, and the denser mesh is used as the slave surface. (2) When the mesh densities of the two contact surfaces are relatively close, the surface with higher stiffness is used as the master surface, and the surface with lower stiffness is used as the slave.

In the definition of the interaction between the contact surfaces, it is also necessary to select the slip algorithm between the contact interfaces according to the actual situation, including small slip and limited slip. If it can be predicted that the slip displacement of the contact surface during the model calculation process is much smaller than the characteristic length of the element (about 0.2 times less than the element size), selecting the small slip algorithm can greatly save the time required for the calculation and improve the computational efficiency. In limited slip, any relative slip can occur between the two contact surfaces. The selection of the finite slip algorithm requires that the main control surface of the contact surface is smooth, that is, each point has a unique normal direction, otherwise the model calculation may not converge. In the calculation process of the finite slip algorithm, it is necessary to constantly determine which part of the slave surface is in contact with the node of the master surface, and the calculation cost is relatively large. For the selection of the slip algorithm between the contact surfaces of the components of the CRTSIII slab ballastless track structure system, when only the vertical static load is considered, the small slip algorithm can be used between the interfaces, but in the case of dynamic loads and lateral forces, it is more reasonable to select the finite slip algorithm.

### 2.2. Selection of Finite Element Model Parameters

The rail in the model is 60 kg/m. The gauge is 1435 mm, which is the standard gauge. The vertical stiffness of the fastener is taken as 30 kN/mm according to the design code [29]. The fastener damping is taken as 0.05 kN·s/mm according to the recommended value in literature [30]. The spacing between adjacent fasteners is 630 mm. The size of the track slab is 5600 mm × 2500 mm × 200 mm. The size of the SCC is 5600 mm × 2500 mm × 90 mm. The size of the base in the subgrade section is 5600 mm × 3100 mm × 300 mm. The thickness of the subgrade bed is 3000 mm, of which the thickness of the surface layer of the subgrade bed is 400 mm and the thickness of the bottom layer of the subgrade bed is 2600 mm.

For the value of friction coefficient in the mutual contact between the layers of the model, according to the field reduction test on the friction coefficient of geotextile in literature [31], the friction coefficient of geotextile is measured to be between 0.75 and 0.9. In the model, the friction coefficient is 0.8, and the friction coefficient between the base and the subgrade is 0.3.

The static wheel load is taken as the uniaxial load. According to the current design speed of a passenger dedicated line and the theoretical research results of ballastless track innovation of a passenger dedicated line, the wheel load is selected as 1.5 times wheel load, i.e., 255 kN [32]. For unit slab ballastless track, slab ends and slab middle can be considered separately when vertical loading is carried out. The values of other parameters of the model are shown in Table 1.

### 2.3. Reliability Verification of Finite Element Model

#### 2.3.1. Verification by Static Load Test

**Figure 5:**
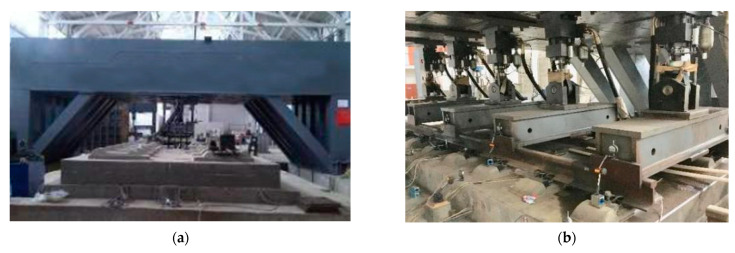

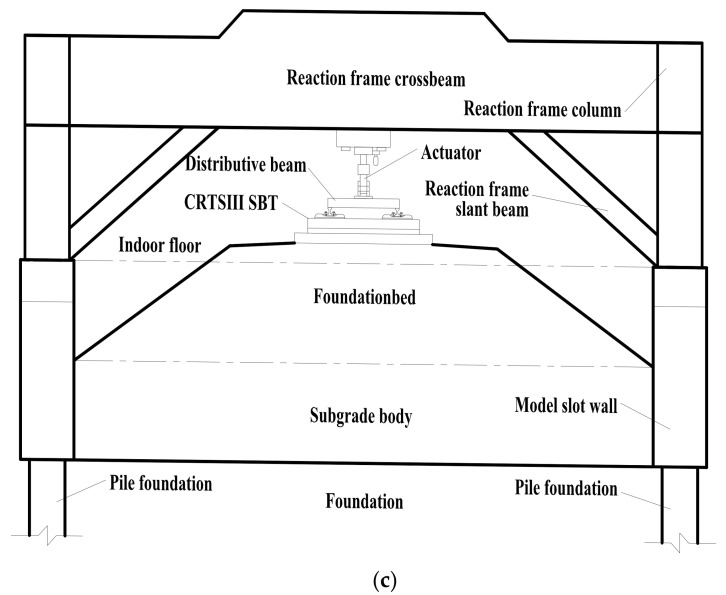
Track–subgrade dynamic model test system: (**a**) model entity diagram; (**b**) model loading diagram; (**c**) cross-sectional view of the model.

**Figure 6:**
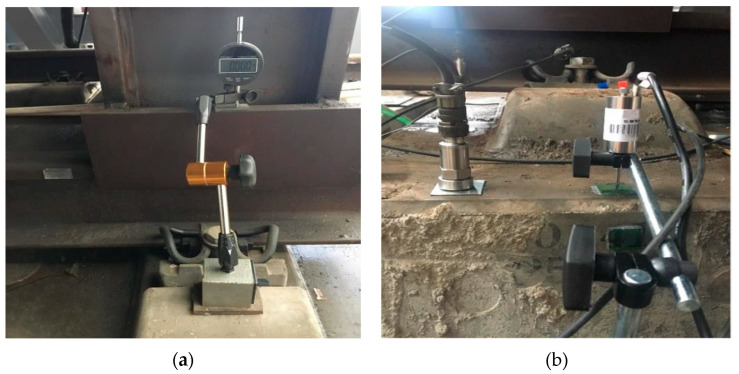
Test components: (**a**) dial indicator; (**b**) accelerometer (**left**) and displacement sensor (**right**).

In order to study the evolution law of fastener stiffness, isolation layer stiffness, and acceleration of various components, our team carried out a 30 million times fatigue test under high-speed train load using CRTSIII SBT subgrade structure full-scale test model and achieved fruitful results [33]. The test model is a standard CRTSIII track, and the corresponding test elements are embedded during the production. The subgrade part of the model adopts the method of on-site filling, and the subgrade model groove is a 16 m × 13 m × 4 m rectangular structure. The track subgrade dynamic model test system after the installation of the track structure is shown in Figure 5, and some details in Figure 5b are shown in Figure 6.

**Figure 7:**
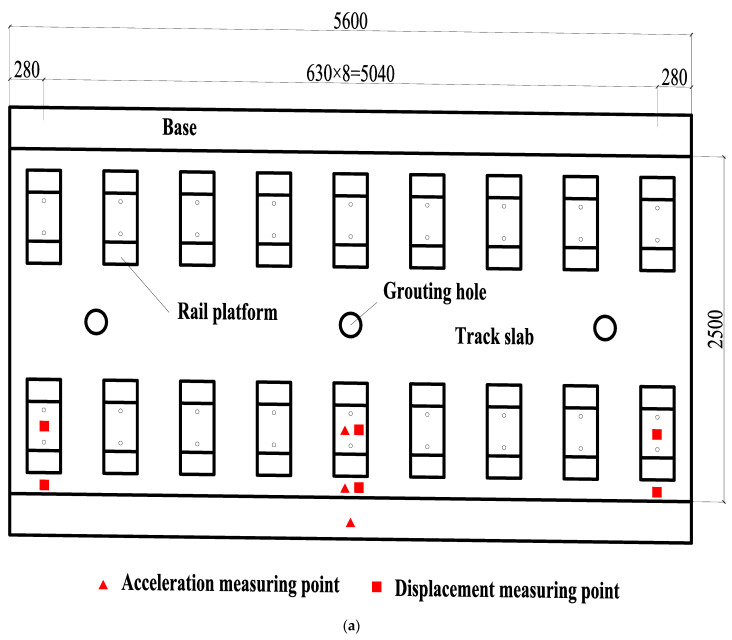

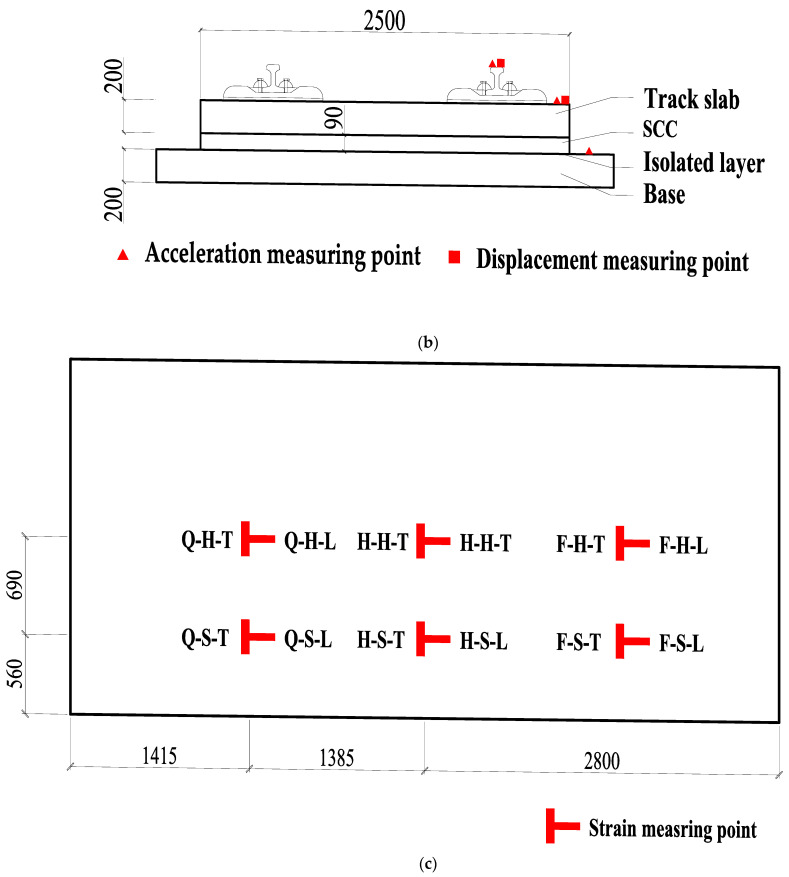
Test instrument layout (unit: mm): (**a**) top view of sensor layout; (**b**) side view of sensor arrangement; (**c**) top view of strain gauge layout.

The static test contents mainly include the relative displacement between rail and track slab, the relative displacement between track slab and base, the longitudinal and transverse strain of track slab concrete, the longitudinal and transverse strain of SCC, the longitudinal and transverse strain of base, etc. The test instruments include a dial indicator and a static strain acquisition instrument. The dynamic test mainly includes the acceleration of rail, track slab, and base; the relative displacement between rail and track slab; and the relative displacement between track slab and base. The test adopts an imc data acquisition instrument for real-time acquisition. The test instruments include displacement sensors and acceleration sensors. The specific layout of the test instruments is shown in Figure 7.

**Figure 8:**
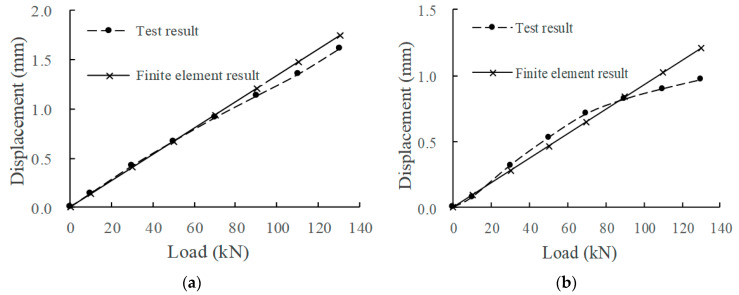
Displacement of rail relative to track slab: (**a**) 0 million times of train load; (**b**) 30 million times of train load.

**Figure 9:**
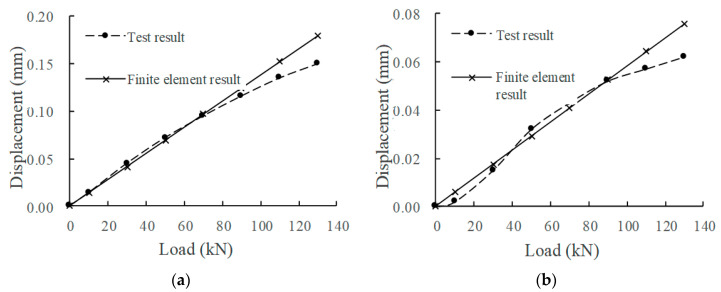
Displacement of track slab relative to base: (**a**) 0 million times of train load; (**b**) 30 million times of train load.

**Figure 10:**
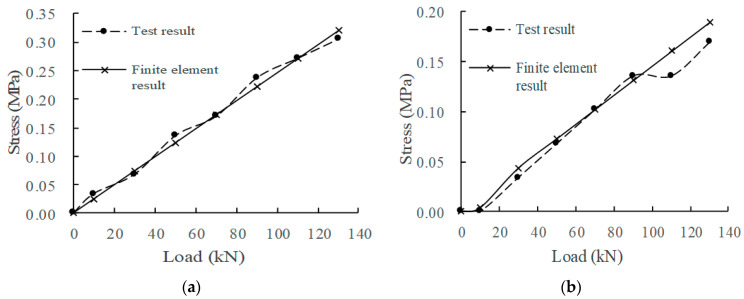
Track slab stress: (**a**) 0 million times of train load; (**b**) 30 million times of train load.

**Figure 11:**
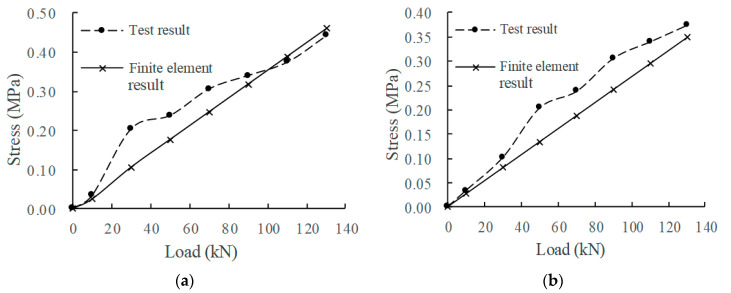
Stress of SCC: (**a**) 0 million times of train load; (**b**) 30 million times of train load.

**Figure 12:**
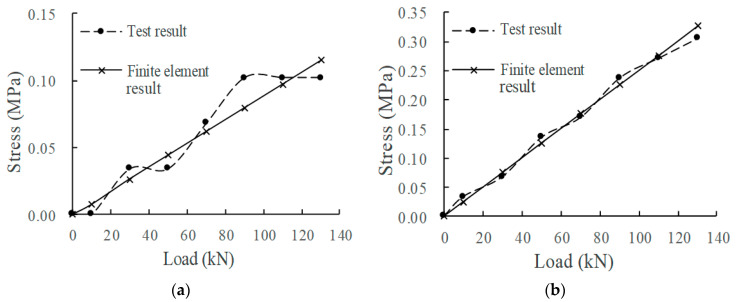
Base slab stress: (**a**) 0 million times of train load; (**b**) 30 million times of train load.

According to the measured data of the mechanical property evolution test of the CRTSIII SBT structure system under train load in literature [33], the test results in the static load graded loading test are compared with the finite element calculation results under the initial state (0 million times of train load) and 30 million times of train load. The comparison indices include the displacement of the rail relative to the track slab, the displacement of the track slab relative to the base slab, and the stress at the corresponding position of each structural layer. The load action times of the finite element model are simulated by changing the fastener stiffness and the stiffness of the isolation layer, as shown in Figure 8, Figure 9, Figure 10, Figure 11 and Figure 12.

Comparing the full-scale model experiment and finite element calculation results before and after fatigue, it can be seen that in terms of stress, the relative error between the calculated value and the experimental value is basically within 20%, the difference is small, and the change trend has a good consistency. As far as displacement is concerned, in the load range of 0~90 kN, the displacement curve calculated by finite element is in good agreement with the experimental displacement curve. Starting from about 90 kN, the two gradually deviate; the slope of the calculated value does not change, but the growth rate of the experimental value slows down. The reason is as follows:

1. Finite element analysis uses a more idealized model to approximate the actual situation. Although the nonlinearity of material properties, geometric nonlinearity, and state nonlinearity are considered in the model calculation, in order to save calculation costs, some noncritical research components are simplified. For example, a wheelset is modeled with discrete rigid bodies. The finite element software is actually an approximate calculation when analyzing nonlinear behavior, so the finite element results will not exactly match the actual situation.
2. When the model is loaded, the loading surface and the bearing surface cannot be absolutely parallel, which aggravates the uneven distribution of stress and displacement of the track structure. Although equipment such as an infrared gradienter is used for leveling during the test, it is impossible to completely eliminate the error caused by the nonparallel condition. In addition, the test environment, data collection, processing of test data, etc., will also affect the authenticity and accuracy of test results.

In general, the finite element analysis curve and the test result curve can be well matched within the error range, and there is little difference with the Hertz theoretical solution. It can be considered that the principle, modeling process, and calculation results of the model are correct and feasible, which is basically consistent with the experimental results.

#### 2.3.2. Reliability Verification of Finite Element Model

The author of [34] conducted axle falling tests at different positions and heights by using a comprehensive test platform of a vehicle line coupling relationship, and simulated the impact load of vehicle wheelset on track. The experimental data have a high reference value for this paper.

The test also focuses on CRTSIII SBT. The full-scale model of the CRTSIII slab ballastless track–subgrade system in the test platform is composed of track and subgrade. The track structure includes steel rail, rail bearing platform, fastening system, track slab, SCC, base, etc. The track slab and the SCC are closely connected through U-shaped ribs, and the SCC adjustment layer is reinforced with single-layer reinforcement mesh. The base slab on the subgrade is continuously poured with reinforced concrete, and two groove retaining platforms are reserved within the same length of the track slab. The longitudinal and transverse forces along the track are transmitted between layers by grooves. The rail used for the test is 60 kg/m, with a gauge of 1.435 m. The length, width, and height of the precast track slab are 5.35 m, 2.5 m, and 0.19 m, respectively. The length and width of the SCC layer are the same as those of the track slab, and the thickness is 0.09 m. The cast in situ base is 3.1 m wide and 0.24 m thick. The rail is fixed on the track slab through a WJ-8B fastener system. The fastener spacing in the slab is 687 mm, the fastener spacing at the slab end is 641 mm, and the slab joint is 100 mm.

**Figure 13:**
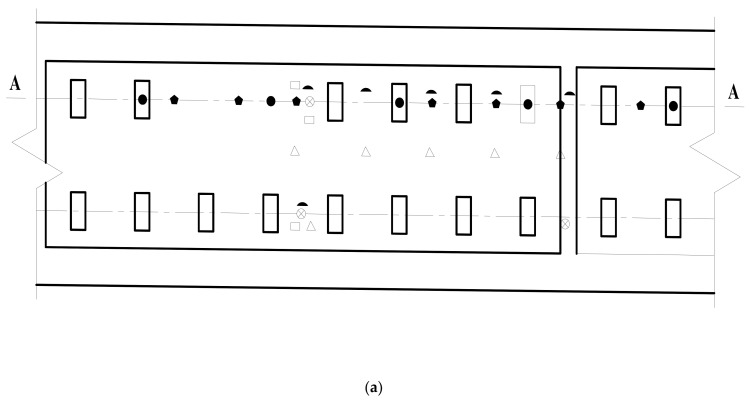

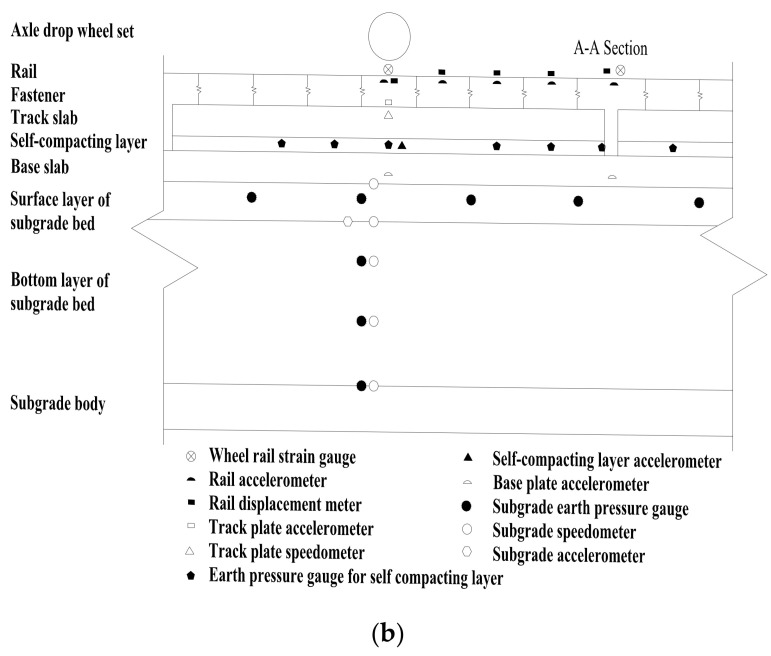
Sensor layout diagram: (**a**) plan view; (**b**) front view.

The sensors arranged in the test mainly include subgrade pressure sensor, subgrade speed sensor, SCC pressure sensor, etc. Among them, the pressure measuring points of the SCC are at the bottom of the layer, that is, the upper surface of the base slab, and their longitudinal positions are set between two adjacent rail bearing platforms. The subgrade pressure sensor depths are 0.2 m, 1 m, 1.8 m, and 2.7 m from the subgrade surface in the direction of depth. The depths from the subgrade speed sensors to the subgrade surface along the depth direction are 0 m, 0.4 m, 1 m, 1.8 m, and 2.7 m. In addition, there is a subgrade acceleration sensor at 0.4 m. The layout of the sensors is shown in Figure 13.

**Figure 14:**
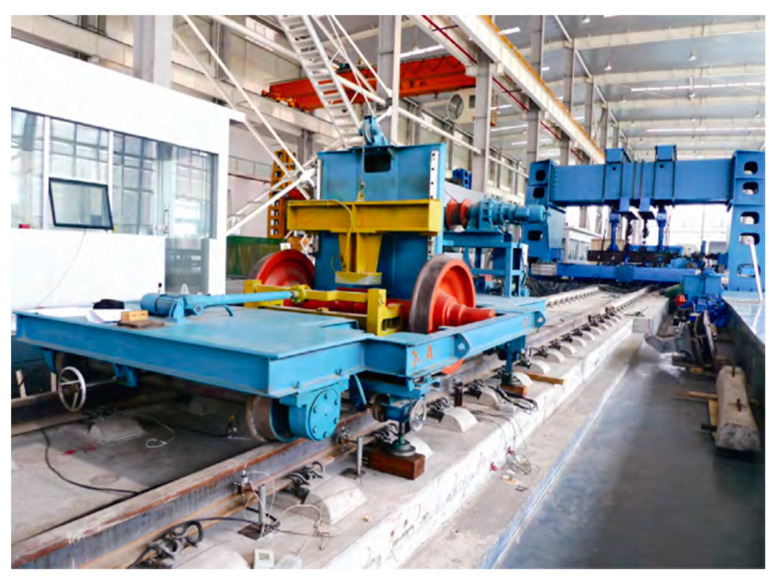
CRTSIII SBT subgrade full-scale model test platform and axle falling impact device.

The loading device for axle falling impact is a railway ballastless track running axle falling test-bed developed by the team with independent intellectual property rights, with a total mass of 4500 kg, including 1020 kg wheelset. The axle falling test vehicle can control the front and rear movement through the remote control device, which is convenient to accurately locate the measuring points and greatly improves the test efficiency and the reliability of the results. Moreover, the safety of the test is high, it is not easy to damage the ballastless track structure, and it is not easy to harm the test personnel. The CRTSIII SBT subgrade full-scale model test platform and axle falling impact device are shown in Figure 14.

The dynamic performance of the finite element structure of the CRTSIII SBT is verified by the falling axle model. The dynamic model inherits the static model and adds a rigid wheelset. The center of mass of the wheelset is taken at the center of the axle, and the mass of the wheelset is converted to 1500 kg according to the equal mass. The axle falling height is set to 20 mm, the axle falling is located in the middle of the slab, and the impact point of axle falling is the transverse midpoint of the rail. Because the load impact time is short enough, only the calculation result of the first 0.05 s is taken.

The numerical calculation results are compared with the measured data of the axle falling test carried out in [34], and the results are shown in Table 2. It can be seen from the table that due to the slight difference between the actual quality of wheelset and the value of track slab structure used in the field test and the model, the impact energy of the track structure in the model calculation is greater than that in the field test, so the calculation result is also slightly larger than that in the field test. However, the overall distribution law of finite element results and field test results is consistent, so the numerical calculation results are reasonable, indicating that the model has good reliability.

## 3. Study on Dynamic Performance of Track Structure under Fatigue State

### 3.1. Analysis of Vibration Transmission Law of Track Structure under Axle Falling Impact

The method of axle falling test can simulate the vertical impact between wheel and rail; measure the response of track structure to impact; and explore the nature, magnitude, and variation law of wheel–rail vertical impact force, so as to optimize the dynamic parameters of track structure and explore the measures to reduce vertical impact [35].

**Figure 15:**
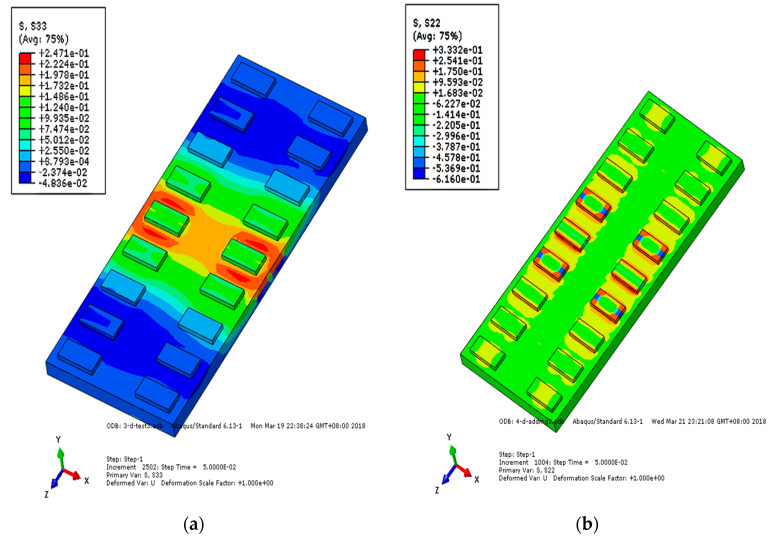
Stress nephogram of track slab under the action of axle falling: (**a**) longitudinal stress of track slab; (**b**) vertical stress of track slab.

**Figure 16:**
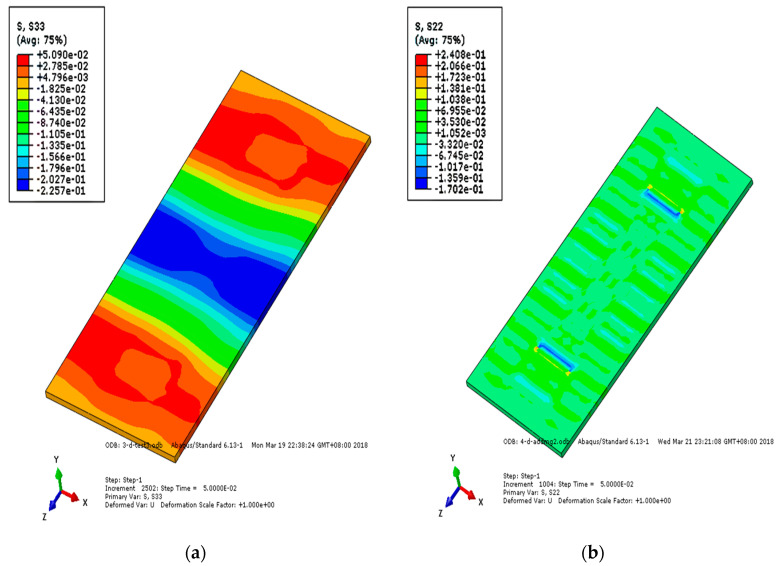
Stress nephogram of SCC under the action of axle falling: (**a**) longitudinal stress of SCC; (**b**) vertical stress of SCC.

**Figure 17:**
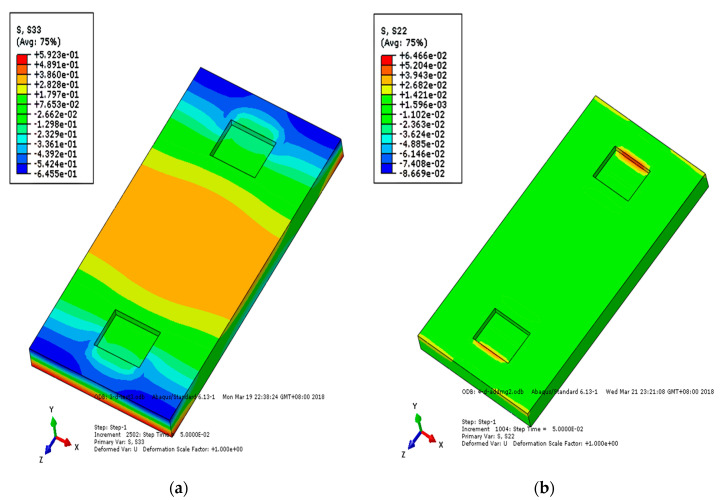
Stress nephogram of substructure under the action of axle falling: (**a**) longitudinal stress of base; (**b**) vertical stress of base.

Under the action of a falling axle, the stress change of each layer of track structure reflects the stress condition of the structure. Figure 15, Figure 16 and Figure 17 show the longitudinal and vertical stress distribution of each layer of track structure after the axle falling impact. It can be seen from the figure that since the CRTSIII SBT structure is a unit slab structure, the impact position of the track structure tends to move upward after the impact. Therefore, along the longitudinal direction of the line, the middle part of the track slab is tensioned and the end is compressed. As the substructure bonded to the track slab, the SCC layer is pressed in the middle and tensioned at the end along the longitudinal direction of the line. As a longitudinal structure, the base is tensioned in the middle and pressed at the end along the longitudinal direction of the line. After the impact, the vertical stress rapidly diffuses into the subgrade structure. Only the rail bearing platform near the action point of the surface layer of the track slab is pulled, and the SCC boss is in contact with the edge of the base groove, resulting in tensile stress and compressive stress, respectively.

### 3.2. Dynamic Response of Track Structure under Fatigue Load

Under the action of the falling axle, the acceleration change of each layer of the track structure reflects the vibration response of the structure under the impact of external load. According to the change of acceleration, the mechanical performance and state of track structure under excitation and the transmission law of the acceleration of the track structure under dynamic action are reflected to a certain extent. The authors of [33] carried out a test on the evolution law of mechanical properties of the CRTSIII SBT slab ballastless track structure under long-term train load. The research shows that the stiffness of the fastener and isolation layer of the track structure increases with the number of loads. The ratio of fastener stiffness and isolation layer stiffness to initial stiffness under 10 million times, 20 million times, and 30 million times of fatigue load are shown in Table 3 [33].

According to the data in Table 3, the fatigue state of track structure is simulated by changing the stiffness of the isolation layer and fastener under the corresponding action times, and the dynamic performance of track structure under fatigue state is analyzed based on the axle falling test. The axle falling height is 20 mm, the axle falling impact point is the transverse midpoint of the rail in the middle of the slab, and the dynamic response changes of each structural layer are shown in Table 4. The rail displacement in the table has been converted into the displacement of rail relative to the track slab. By analyzing the data in the table, the following observations can be made:

1. With the increase in fatigue load time, the wheel–rail force increases, the rail displacement decreases, the acceleration of rail and base increases obviously, but the acceleration of track slab changes little. The reason is that the fatigue load leads to the decline in the vibration damping performance of the isolation layer, and the action time between the axle and the rail decreases, so the acceleration of the base and the acceleration track slab gradually converge.
2. With the increase in fatigue load times, the surface tensile stress of SCC decreases, while the surface compressive stress of base increases, and the two tend to be stable after 20 million loading times. The longitudinal stress on the surface of the track slab is basically unchanged.
3. With the increase in the number of fatigue loads, the vertical stress of each layer of the track structure increases, and the vertical stress of the base increases most significantly, increasing by more than 10%.

## 4. Study on Dynamic Performance of Track Structure under Interface Parting Condition

The CRTSIII SBT structure will exhibit interface parting under the actual service state. Due to the diversity and randomness of positions, the parting conditions are also different, and the separation methods are also divided into many kinds. Through the field investigation and statistics of various parting conditions (including the longitudinal and transverse development length of the parting along the line, as well as the parting position and other factors), the parting between track slab and SCC can be roughly divided into the following four categories according to the position: parting under the edge of the track (TEP), parting under the rail (RP), parting under the middle of the track (TMP), and complete parting (CP) [36]. Based on the axle falling test simulation, this section analyzes the dynamic performance of the track structure under the conditions of TEP, RP, TMP, and CP, mainly studying the acceleration, speed, displacement, longitudinal stress, and vertical stress of each structural layer. The height of axle falling is 20 mm, and the impact point of axle falling is the transverse midpoint of the rail in the middle of the slab. The dynamic response of each structural layer at the position directly below the axle falling point is analyzed, and the main research objects are the acceleration, velocity, displacement, longitudinal stress, and vertical stress of each structural layer.

### 4.1. Study on Dynamic Performance of Track Structure in the Condition of TEP

**Figure 18:**
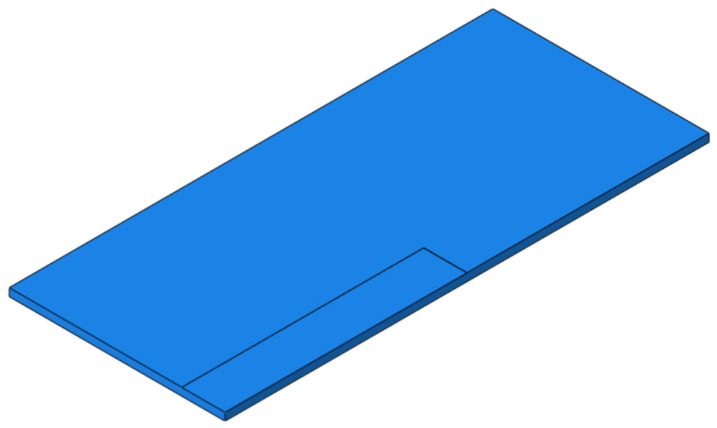
The TEP of SCC.

For the TEP, the width of SCC is set to 500 mm and the height to 2 mm, and the seam starts from the corner of the slab and develops longitudinally along the line to the middle of the slab, as shown in Figure 18. The dynamic performance of the track structure under the condition is analyzed.

**Figure 19:**
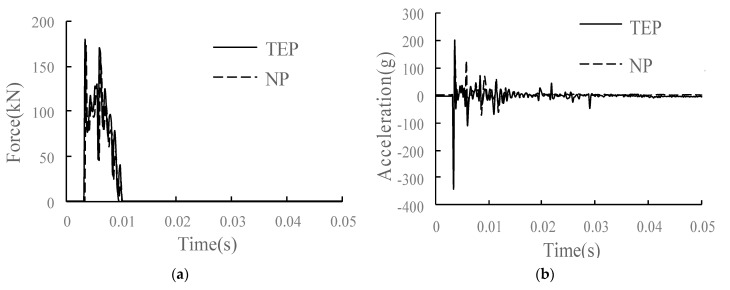

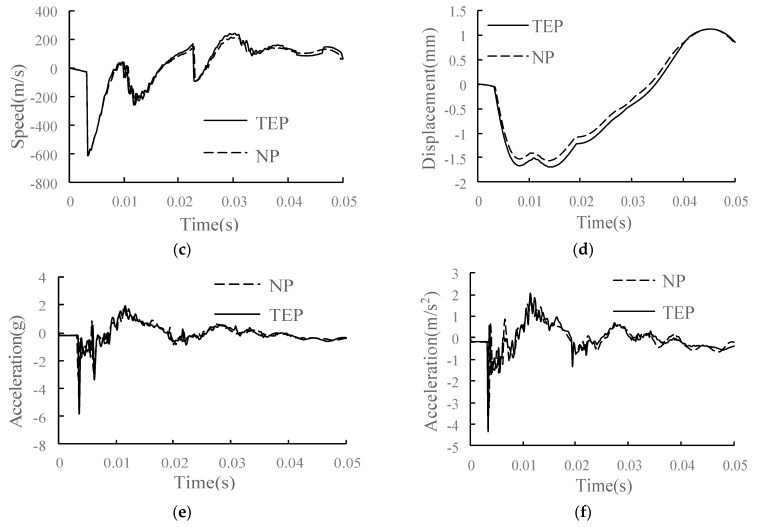

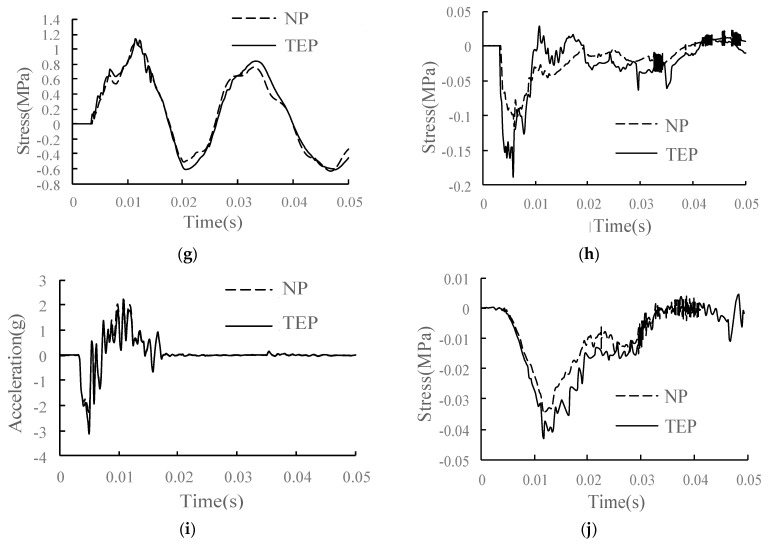
Dynamic response diagram of each structural layer in the condition of TEP: (**a**) wheel–rail vertical force; (**b**) rail acceleration; (**c**) rail vertical speed; (**d**) rail vertical displacement; (**e**) track slab acceleration; (**f**) acceleration of SCC layer; (**g**) longitudinal stress of SCC layer; (**h**) vertical stress of SCC layer; (**i**) base acceleration; (**j**) vertical stress of substructure.

The comparison of the main dynamic response time history curves of each structural layer in the conditions of TEP and no parting (NP) is shown in Figure 19. Compared with the condition of NP, in the condition of TEP, the overall vibration response of the track structure at the parting side is increased due to the weakening of support strength and structural constraints. For the wheel–rail vertical force, it increases slightly, with an increase of 4.5%. For the rail, the vertical speed of the rail is almost unchanged; the rail displacement increases; the rail acceleration increases most significantly, with an increase of 28.5%; and the rail vibration intensifies significantly. The acceleration and vertical stress of the track slab and SCC layer change significantly. The acceleration of the track slab and acceleration of the SCC layer increase by 87.5% and 33.2%, respectively, and the vertical stress of the SCC layer increases by 72.7%. For the base and subgrade bed, due to the damping effect of the isolation layer, the changes of acceleration and vertical stress are relatively weakened, the acceleration of the base increases by 23%, and the dynamic response of the surface layer of the subgrade bed has little change.

### 4.2. Study on Dynamic Performance of Track Structure in the Condition of RP

**Figure 20:**
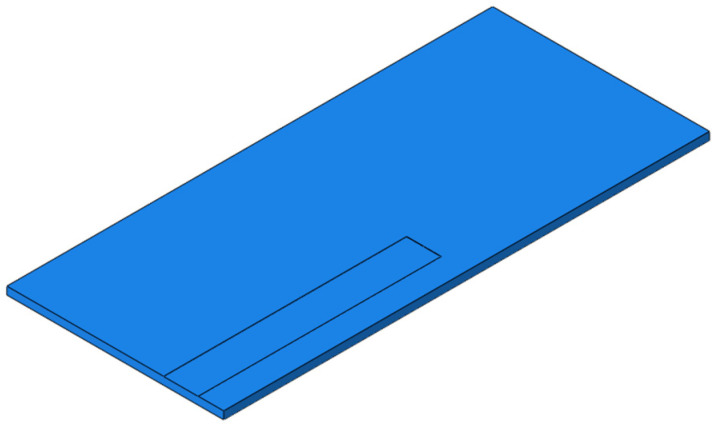
The RP of SCC.

For the RP, the width of SCC is set to 400 mm and the height to 2 mm, and the parting starts from the position below the rail of the slab and develops longitudinally along the line to the middle of the slab, as shown in Figure 20. The dynamic performance of the track structure under the condition is analyzed.

**Figure 21:**
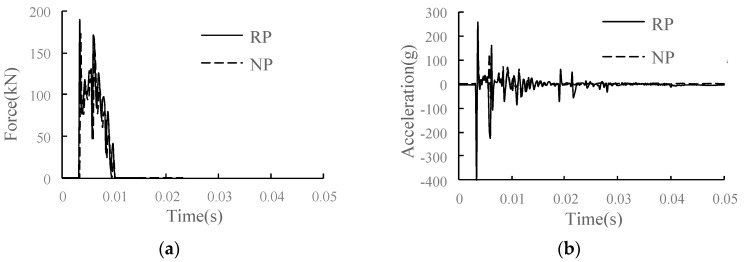

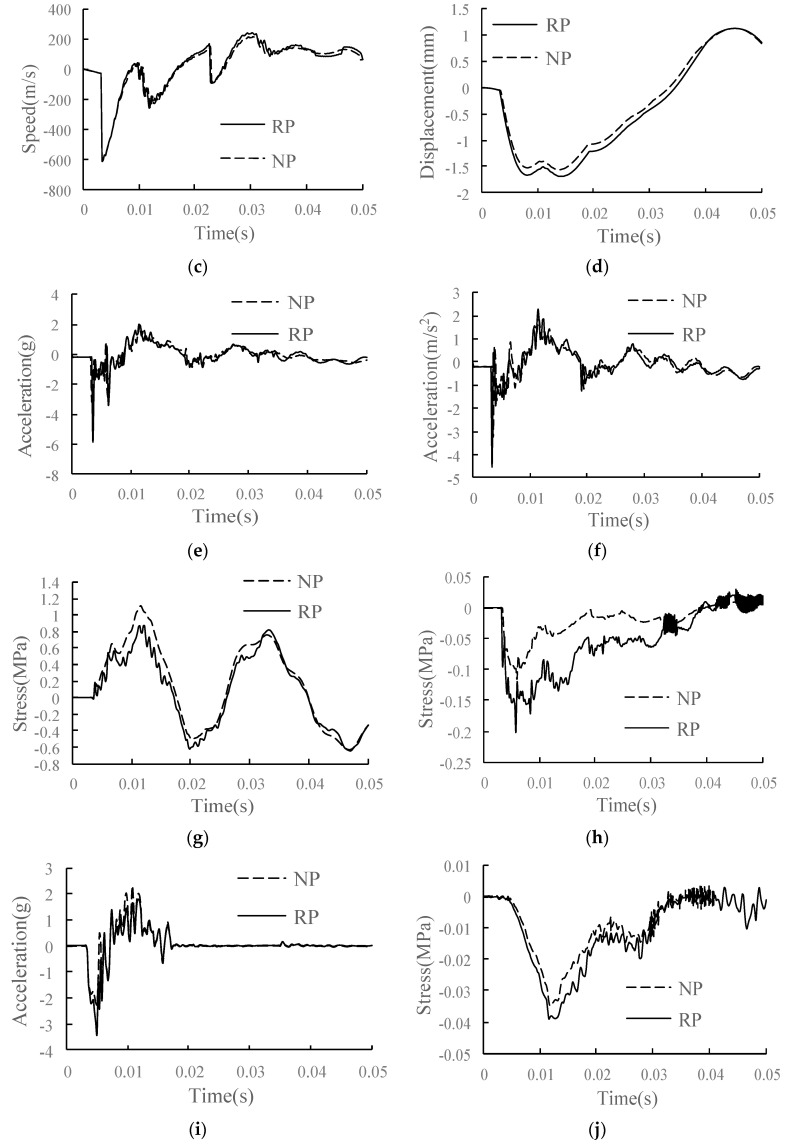
Dynamic response diagram of each structural layer in the condition of RP: (**a**) wheel–rail vertical force; (**b**) rail acceleration; (**c**) rail vertical speed; (**d**) rail vertical displacement; (**e**) track slab acceleration; (**f**) acceleration of SCC layer; (**g**) longitudinal stress of SCC layer; (**h**) vertical stress of SCC layer; (**i**) base acceleration; (**j**) vertical stress of substructure.

The comparison of the main dynamic response time history curves of each structural layer in the conditions of RP and NP is shown in Figure 21. Compared with the condition of NP, in the condition of RP, the parting area increases the overall vibration response of the track structure due to the weakening of support strength and structural constraints. The wheel–rail vertical force is increased by 10.5%. The wheel–rail force obviously increases on the parting side due to the damage of the structure under the rail. For the rail, the vertical speed of the rail is almost unchanged; the rail displacement increases, with an increase of 12.5%; and the rail acceleration increases most significantly, with an increase of 50.4%. The rail vibration increases significantly when the rail is separated from the joint under the rail. The speed of the track slab and SCC layer changes little, the displacement increases slightly, and the acceleration and vertical stress change significantly. Acceleration increases of track slab and SCC layer are 87.5% and 39.1%, respectively. As the parting develops to the rail–falling axle interface, the longitudinal stress of the SCC layer decreases by 22.2%, the vertical stress increases by 81.8%, and the dynamic response of the SCC layer changes sharply. For the base and subgrade bed, due to the damping effect of the isolation layer, the changes of acceleration and vertical stress are relatively weakened, the acceleration of the base increases by 32.7%, and the dynamic response of the surface layer of the subgrade bed has little change.

### 4.3. Study on Dynamic Performance of Track Structure in the Condition of TMP

**Figure 22:**
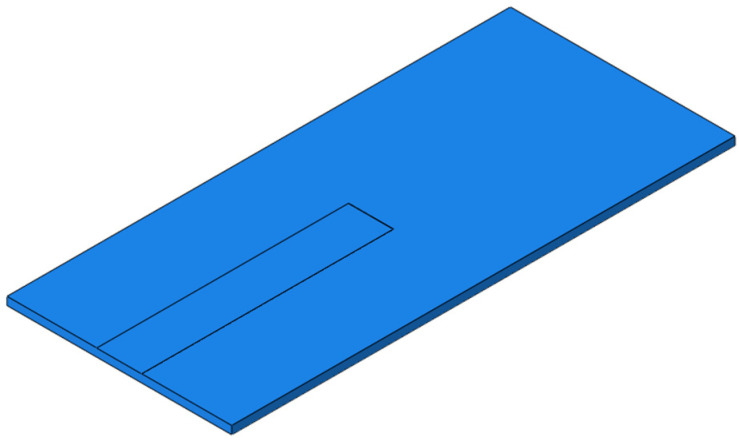
The TMP of SCC.

For the TEP, the width of SCC is set to 500 mm and the height to 2 mm, and the parting develops from the position between the two rails at the slab end along the longitudinal direction of the line to the middle of the slab, as shown in Figure 22. The dynamic performance of the track structure under the condition is analyzed.

**Figure 23:**
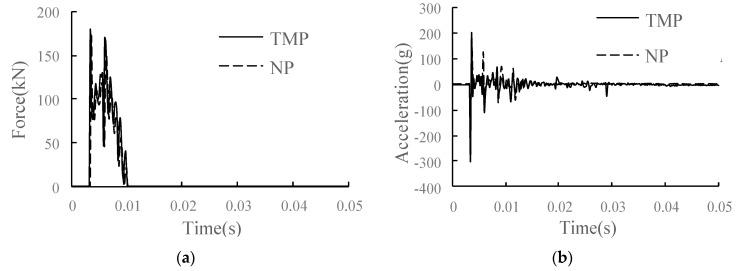

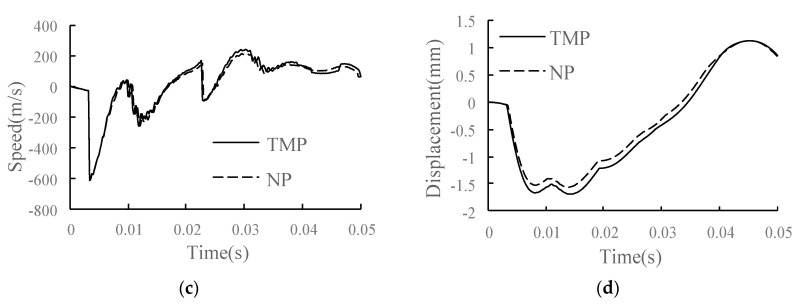

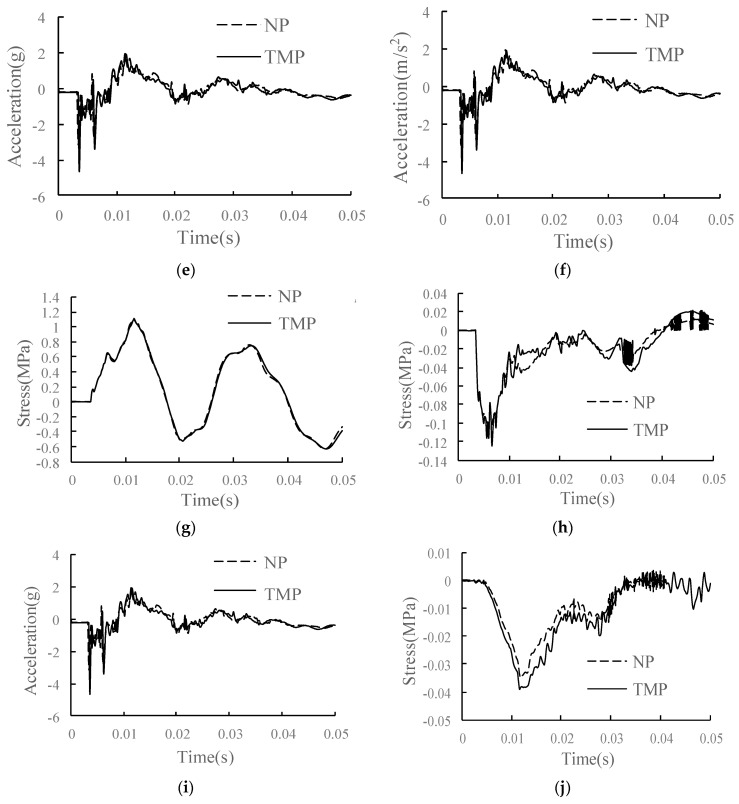
Dynamic response diagram of each structural layer in the condition of TMP: (**a**) wheel–rail vertical force; (**b**) rail acceleration; (**c**) rail vertical speed; (**d**) rail vertical displacement; (**e**) track slab acceleration; (**f**) acceleration of SCC layer; (**g**) longitudinal stress of SCC layer; (**h**) vertical stress of SCC layer; (**i**) base acceleration; (**j**) vertical stress of substructure.

The comparison of main dynamic response time history curves of each structural layer in the conditions of TMP and NP is shown in Figure 23. Compared with the condition of NP, in the condition of TMP, the overall vibration response of the track structure at the parting side is increased due to the weakening of support strength and structural constraints. For the wheel–rail vertical force, it increases slightly, with an increase of 3.4%. For the rail, the vertical speed of the rail is almost unchanged; the rail displacement increases; the rail acceleration increases most significantly, with an increase of 12.7%; and the rail vibration intensifies. For track slab and SCC, the speed changes little, the displacement and longitudinal stress increase slightly, and the acceleration and vertical stress change significantly. The acceleration increase in the track slab and SCC layer is 36.9% and 17.8%, respectively, and the vertical stress increase in the SCC layer is 36.3%. The dynamic response of the SCC layer changes sharply. For the base and subgrade bed, due to the damping effect of the isolation layer, the changes of acceleration and vertical stress are relatively weakened, the acceleration of the base increases by 13.2%, and the dynamic response of the surface layer of the subgrade bed has little change.

### 4.4. Study on Dynamic Performance of Track Structure in the Condition of CP

**Figure 24:**
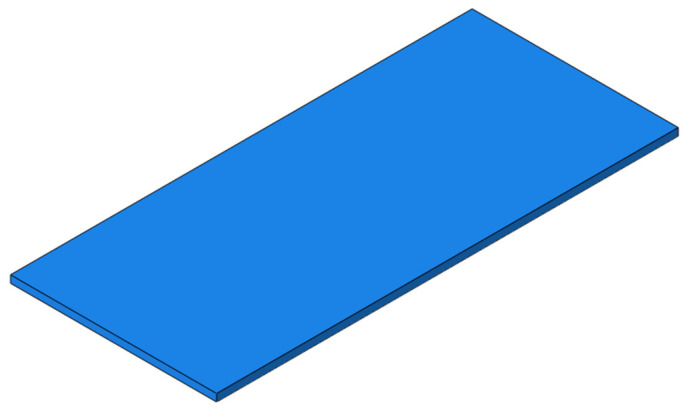
The CP of SCC.

For the CP, the joint between the SCC layer and the track slab is completely separated, and the whole interface is connected only by the U-shaped ribs, with a parting height of 2 mm, as shown in Figure 24. The dynamic performance of the track structure under the condition is analyzed.

**Figure 25:**
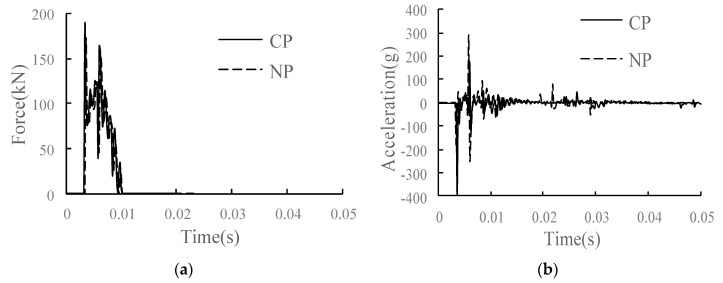

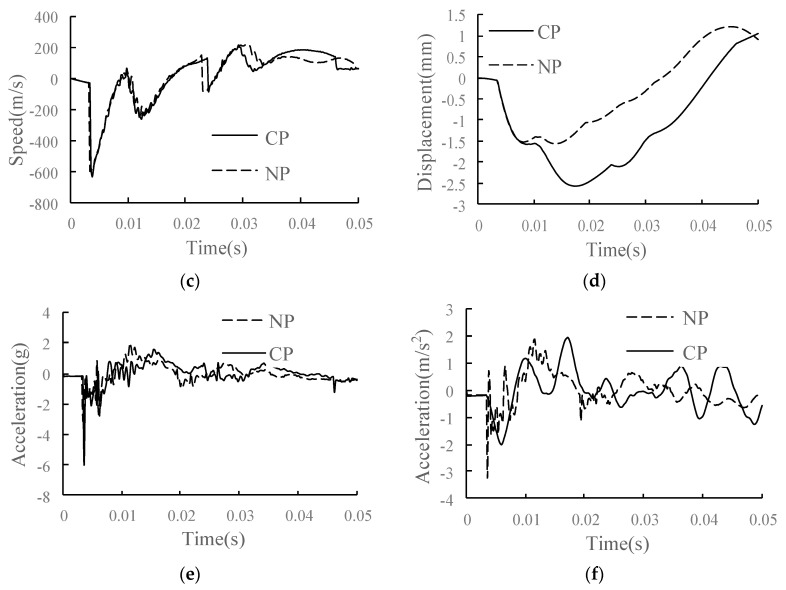

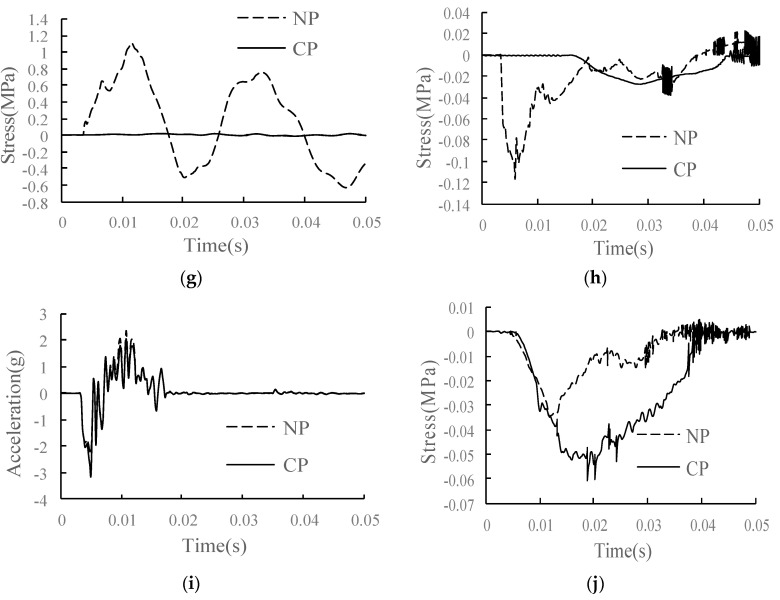
Dynamic response diagram of each structural layer in the condition of CP: (**a**) wheel–rail vertical force; (**b**) rail acceleration; (**c**) rail vertical speed; (**d**) rail vertical displacement; (**e**) track slab acceleration; (**f**) acceleration of SCC layer; (**g**) longitudinal stress of SCC layer; (**h**) vertical stress of SCC layer; (**i**) base acceleration; (**j**) vertical stress of substructure.

The comparison of the main dynamic response time history curves of each structural layer in the conditions of CP and NP is shown in Figure 25. Compared with the condition without joint separation, under the condition of complete joint separation of the interface, the track slab and the SCC layer are connected by U-shaped ribs instead of closely bonded composite structure, which increases the overall vibration response of the track structure. The wheel–rail vertical force increased by 10.4%. For the rail, the vertical speed of the rail does not change significantly, and the acceleration and displacement of the rail increase significantly, with increases of 51.2% and 68.4%, respectively. The rail vibration increases significantly. The dynamic response of the track slab and SCC layer changes significantly. Acceleration of track slab increases by 93.6%, and the longitudinal and vertical stresses increase by 32.2% and 7.3%, respectively. Due to the loss of bonding between the surface layer of the SCC and track slab, the stress is transmitted only through U-shaped ribs, so the acceleration and longitudinal and vertical stresses of the SCC layer are greatly reduced. For the base and subgrade bed, the vertical stress of the base increases by 64.7%, the acceleration and longitudinal stress decrease, and the dynamic response of the surface layer of the subgrade bed changes little.

## 5. Conclusions

In this paper, the finite element model of the CRTSIII SBT structure is established to verify the reliability of its static load and axle falling simulation. Through the axle falling simulation of different fatigue load time and parting conditions, the vibration transmission law of the track structure and the change of dynamic force response of each layer are analyzed, and the following conclusions can be drawn:

1. As the CRTSIII SBT structure is a unit slab structure, the impact position tends to move upward after the impact of axle falling. Therefore, along the longitudinal direction of the line, the middle part of the track slab is tensioned and the end is compressed; as the substructure bonded to the track slab, the SCC layer is compressed in the slab and tensioned at the slab end; as a longitudinal structure, the base is tensioned in the surface layer of the base and compressed at the slab end. After the impact, the vertical stress diffuses rapidly into the subgrade structure; only the rail bearing platform near the action point of the surface layer of the track slab is tensioned, and the SCC boss is in contact with the edge of the base groove, resulting in tensile and compressive stresses, respectively.
2. Under the fatigue load, the acceleration of rail and base increases obviously, and with the weakening of vibration damping performance of the isolation layer, the acceleration of base and the acceleration of the track slab gradually converge. The longitudinal tensile stress of the SCC surface decreases and that of the base surface increases, while the vertical stress of each layer of track structure increases. The vertical stress of the base increases the most, reaching 44.9%, which is unfavorable to the base structure.
3. The dynamic response of each structural layer of the track increases when the interface is partially separated. The acceleration and stress changes of each layer are the most obvious under the point of falling of the axle. Because of falling of the axle, the longitudinal tensile stress of the SCC layer even decreases, and the uneven development of tensile stress easily leads to further development of the parting.
4. In the condition of CP, the track slab and the SCC layer are changed from a closely bonded composite structure to being only connected by U-shaped ribs. The acceleration and longitudinal and vertical stresses of the SCC layer are greatly reduced, and the speed and displacement are increased; the acceleration and displacement of the track slab are greatly increased, and the dynamic response of the track structure is significantly increased.

## Figures and Tables

**Table 1:** Parameters of CRTSIII SBT structure model.

Structural Components	Performance Parameter	Numerical Value
Rail	Elastic modulus (MPa)	2.06 × 10^5^
	Density (kg/m^3^)	7.80 × 10^3^
	Poisson’s ratio	0.3
Fastener	Stiffness (kN/mm)	30 × 10^3^
	Damping (kN·s/mm)	0.05
Track slab	Elastic modulus (MPa)	3.60 × 10^4^
	Density (kg/m^3^)	2.50 × 10^3^
	Poisson’s ratio	0.17
SCC	Elastic modulus (MPa)	3.4 × 10^4^
	Density (kg/m^3^)	2.40 × 10^3^
	Poisson’s ratio	0.2
Base	Elastic modulus (MPa)	3.2 × 10^4^
	Density (kg/m^3^)	2.50 × 10^3^
	Poisson’s ratio	0.2
Surface layer of subgrade bed	Elastic modulus (MPa)	300
	Density (kg/m^3^)	1.95 × 10^3^
	Poisson’s ratio	0.3
Bottom layer of subgrade bed	Elastic modulus (MPa)	250
	Density (kg/m^3^)	1.90 × 10^3^
	Poisson’s ratio	0.25

**Table 2:** Dynamic response of each structural layer under the action of axle falling.

Index	Location	Paper Model	Literature [34] Test Value
Acceleration (g)	Rail	261.3	239.2
	Track slab	3.30	3.14
	Base	2.55	2.1
Speed (mm/s)	Rail	610.2	601.8
	Track slab	29.1	28.0
	Base	17.6	—

**Table 3:** Ratio of fastener and isolation layer stiffness to the initial under different load action times.

Index	10 Million Times	20 Million Times	30 Million Times
Fastener stiffness	1.15	1.26	1.29
Isolation layer stiffness	1.78	2.18	2.44

**Table 4:** Comparison of maximum dynamic response of track structure surface under axle falling.

Index	Location	The Initial State	10 Million Times	20 Million Times	30 Million Times
Wheel–rail force (kN)	Wheel–rail interface	170.1	177.3	180.1	179.9
Speed (mm/s)	Rail	611.2	614.2	629.8	646.5
Displacement (mm)	Rail	1.52	1.48	1.44	1.39
Acceleration (g)	Rail	262.7	321.3	341.7	353.6
	Track slab	3.11	3.10	3.14	3.16
	Base	2.57	2.79	2.94	3.03
Longitudinal stress (MPa)	Track slab	−1.01	−1.02	−1.03	−1.04
	SCC	1.17	1.06	1.01	1.01
	Base	−1.37	−1.46	−1.48	−1.49
Vertical stress (MPa)	Track slab	−0.46	−0.50	−0.51	−0.52
	SCC	−0.11	−0.12	−0.13	−0.14
	Base	−0.0372	−0.042	−0.0485	−0.0539

## Data Availability

Experimental data have been presented in the text.
